# Detection of fungal and bacterial carbohydrates: Do the similar structures of chitin and peptidoglycan play a role in immune dysfunction?

**DOI:** 10.1371/journal.ppat.1007271

**Published:** 2018-10-11

**Authors:** Jonathan Dworkin

**Affiliations:** Department of Microbiology and Immunology, College of Physicians and Surgeons, Columbia University, New York, New York, United States of America; McGill University, CANADA

## Introduction

**Fig 1:**
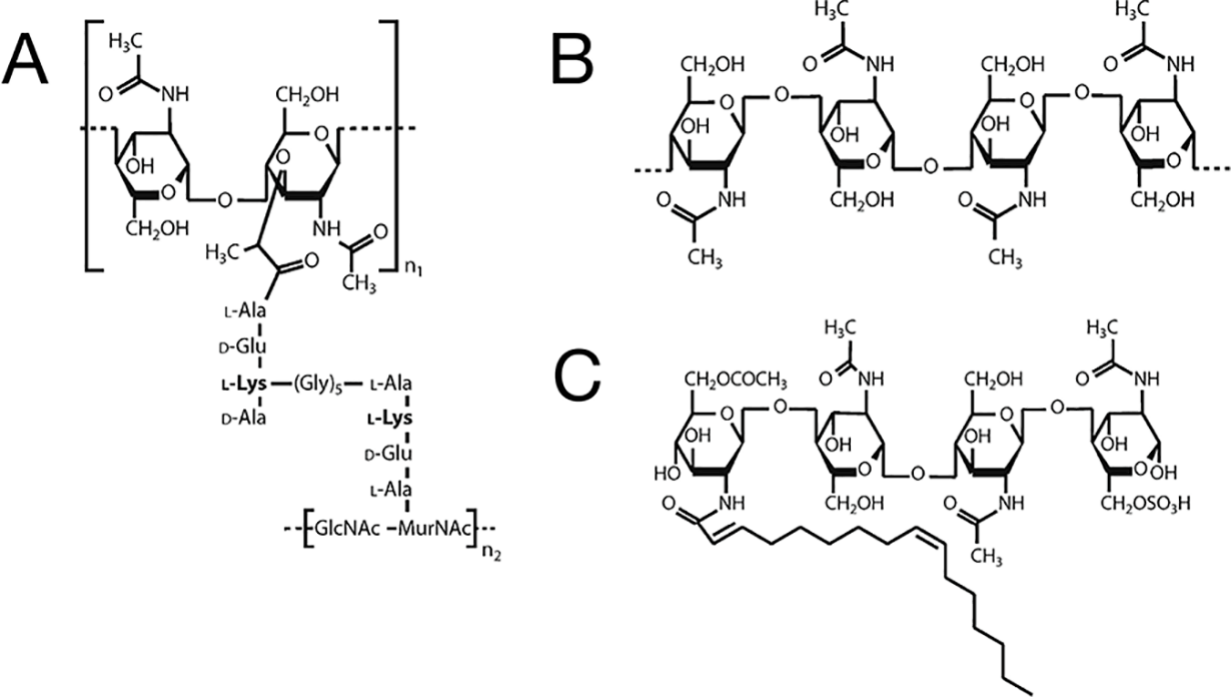
Bacterial and fungal carbohydrates. A. Monomer of L-Lys containing peptidoglycan from *Staphlycoccus aureus*. B. Fungal chitin. C. *Sinorhizobium meliloti* Nod factor NodSmIV. Lys, Lysin; Nod, Nodulation.

Carbohydrate recognition is fundamental to a wide variety of interkingdom interactions. For example, bacterial peptidoglycan, an *N*-acetyl-D-glucosamine (GlcNAc)–*N*-acetylmuramic acid (MurNAc) polymer [1], and fungal chitin, a GlcNAc polymer [2, 3], are both immunostimulatory to vertebrates. In addition, bacterially produced Nodulation (Nod) factors, which consist of a modified GlcNAc, play a key role in symbiotic interactions with plants [4]. The structural similarity of these GlcNAc-containing molecules (Fig 1) suggests possible overlap in their physiological action, although many of the mechanisms underlying the detection of these molecules in different organisms appear unrelated. However, a single phylogenetically conserved domain, called Lysin (LysM), is found in specific receptors in signaling pathways responsive to one or more of these three related carbohydrates in bacteria, plants, and fungi and possibly mammalian systems. Thus, promiscuous activation could occur when a structurally similar but physiologically inappropriate ligand binds and thereby aberrantly activates an incorrect LysM domain-containing receptor. Here, I will discuss this possibility and its implications for immune pathologies such as asthma in which chitin is relevant.

### Bacterial carbohydrates: Peptidoglycan

Most bacteria have cell walls containing peptidoglycan, a polymer composed of alternating β(1,4)-linked GlcNAc and MurNAc residues cross-linked by species-specific peptides (Fig 1A). Many proteins are attached noncovalently to the bacterial cell envelope, and the LysM motif is often used to target these proteins to the peptidoglycan. LysM domains are found in a wide range of bacterial proteins that are involved in cell wall metabolism, including peptidoglycan hydrolases and phage lysins [5]. Detailed structural analysis of a LysM domain from a bacterial peptidoglycan hydrolase reveals that it recognizes both the GlcNAc moiety as well as the peptide stem [6]. In plants, LysM domain-containing proteins serve as peptidoglycan receptors and initiate a downstream signaling cascade in response to peptidoglycan binding [7]. While a number of different mammalian proteins are known to recognize peptidoglycan, such as Peptidoglycan recognition proteins (PGRP) [8], they lack LysM motifs. Interestingly, there are recently identified LysM domain-containing proteins in vertebrates, including zebrafish [9] and mice [10], although their physiological function is not known.

### Fungal carbohydrates: Chitin

Chitin, a linear β(1,4)-linked polymer of GlcNAc (Fig 1B) derived from a variety of biological sources including the cell walls of fungi and insects, is one of the most abundant polymers in nature. In plants, chitin is sensed by LysM domain-containing proteins that function to mediate an appropriate innate response [11]. In fungi, LysM domain-containing proteins called LysM effectors are important for chitin-triggered immunity, possibly by acting quite generally on processes including spore germination and hyphal growth [12]. Chitin (and its deacetylated form chitosan) are immunostimulatory in mammals, although the mechanism underlying chitin recognition remains elusive, perhaps in part because variables including the size of the physiologically active chitin molecules are not known [3].

### Symbiotic carbohydrates: Lipochitin oligosaccharides

The symbiotic relationship between legumes and rhizobial bacteria is central to biological nitrogen fixation and thereby to the global nitrogen cycle. Key to this relationship are Nod factors, short species-specific chito-oligosaccharides containing various substitutions on the reducing and nonreducing termini. One example of a Nod factor is NodSmIV, produced by *Sinorhizobium meliloti* (Fig 1C), and as can be seen, it is very similar to both peptidoglycan and chitin. The plant Nod factor receptors are receptor kinases that contain one to three extracellular LysM domains [13] with very high (approximately nM) affinity for their specific Nod factor ligand [14].

### Potential cross-reactivity in peptidoglycan and chitin recognition

In fungi, LysM domain-containing proteins called LysM effectors play a role in regulating host colonization, possibly by sequestering fungal-derived chitin [12]. It has been proposed that the ability of LysM domains to also bind peptidoglycan may allow fungi to affect potential bacterial competitors during infection [12]. This could occur because cell wall binding even in the absence of muralytic activity can be sufficient for antibacterial function [15]. In plants, the LysM domains that mediate Nod factor recognition are also found in other proteins that serve as receptors for pathogenic signals including chitin and/or peptidoglycan [16, 17]. This has led to the intriguing hypothesis that Nod factors may have evolved as a mechanism to suppress innate immunity by interfering with the recognition of chitin and/or peptidoglycan [18]. In support of this idea, Nod factors suppress an innate immune reaction in *Arabidopsis thaliana*, even though this species lacks a Nod receptor [14]. Since this effect depends on the presence of the LYK3 LysM-containing receptor kinase that functions as an innate immune receptor, the Nod factor could therefore act as a competitive antagonist of LYK3.

**Fig 2:**
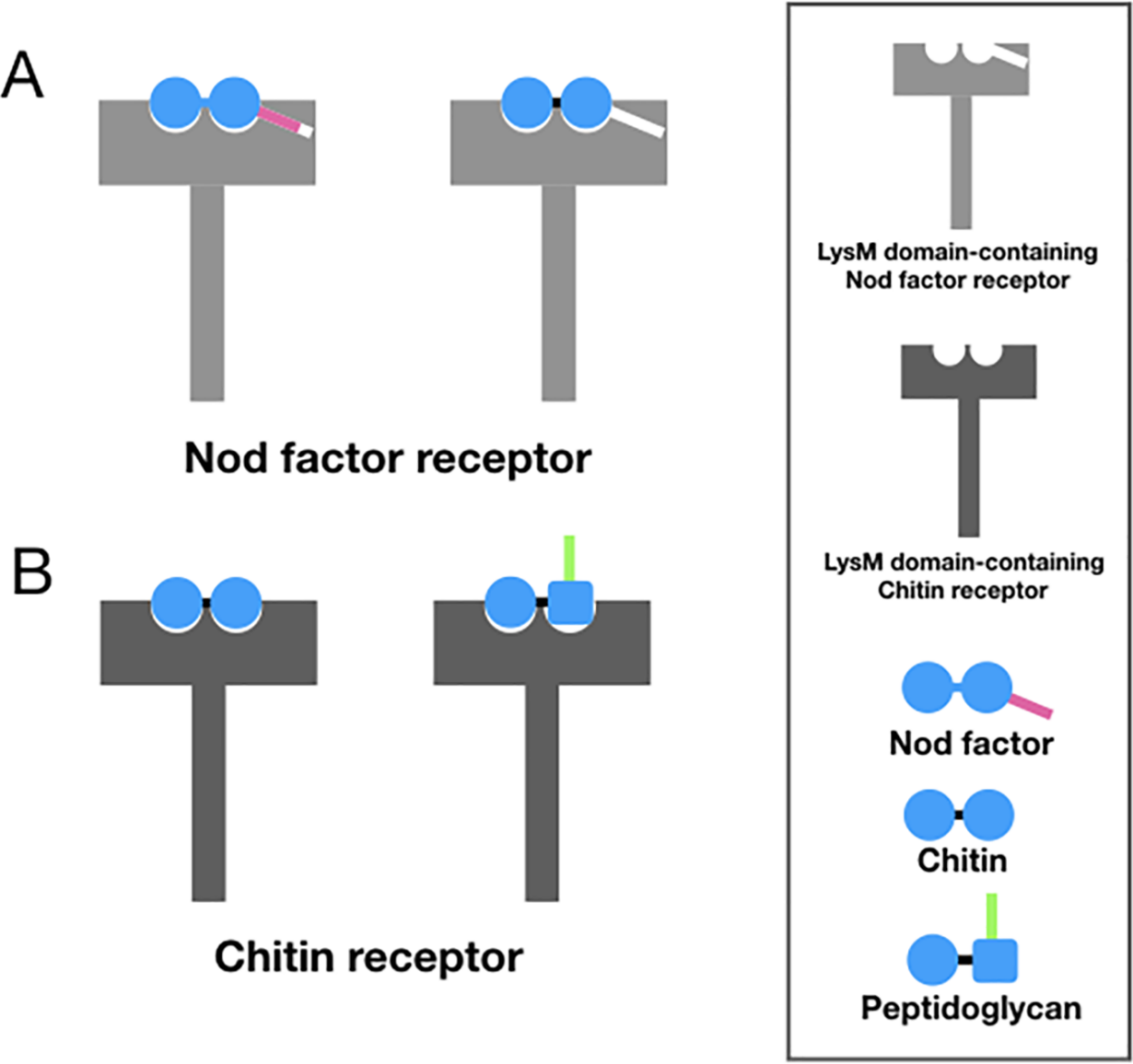
Potential cross-activation of LysM domain-containing carbohydrate receptors. A. The LysM domain of a Nod factor receptor normally binds Nod factor (left) but could also bind a structurally related chitin molecule (right). B. The LysM domain of a chitin receptor that normally binds chitin (left) could also bind a structurally similar peptidoglycan muropeptide (right). Lys, Lysin, Nod; Nodulation.

In the case of plants, the ability of LysM domain-containing proteins to bind both chitin and Nod factors presents a potential problem, given the heterogeneous mixture of microbially produced carbohydrates present in the soil. Specificity of the physiological response could be maintained through distinct receptor sets, which have a greater affinity for one class of saccharide. Recently, such a mechanism has been shown to operate in the legume *Lotus japonicus*, in which a LysM domain-containing kinase that mediates the response to pathogens exhibits much higher sensitivity to chitin as compared to Nod factors [19]. In mammalian cells, the ability of Nod factors (lipochitin oligosaccharides) to stimulate mammalian angiogenesis [20], taken with the previously mentioned observation that a Nod factor is able to suppress a plant innate immune reaction in species that lack the Nod receptor [14], suggests that physiologically relevant cross-activation is possible. For example, a chitin derived molecule could bind a LysM domain that normally binds Nod factor (Fig 2, left). Conversely, a peptidoglycan-derived muropeptide could bind a LysM domain that normally binds chitin (Fig 2, right). Presumably, these noncognate bindings would occur at relatively lower affinity, but if environmental concentrations of the heterologous ligands are in excess to the normal ligand, then noncognate binding could be physiologically relevant.

So what direct evidence exists that particular LysM domains, which mediate detection of either peptidoglycan or chitin in a particular physiological context, could promiscuously interact with both ligands? That is, could chitin or peptidoglycan interact with a LysM domain of a protein that is normally the receptor for the other molecule? The structure of *Enterococcus faecalis* AtlA, a LysM domain-containing peptidoglycan hydrolase, indicates that specific, well-conserved residues in the AtlA LysM domain mediate binding to GlcNAc-(GlcNAc/MurNAc)-GlcNAc. This structure implies that further specificity is determined by secondary interactions between hydrophobic LysM residues such as leucine that interacts with hydrophobic substitutions of the nonreducing terminal sugar in the case of Nod factor recognition or the interaction mediated by a nonaromatic residue at the reducing end of the ligand in the case of peptidoglycan [6].

Both in vitro and in vivo evidence supporting some promiscuity exists (Table 1). In vitro, *E*. *faecalis* AtlA binds chitin with a higher affinity than peptidoglycan [6]. LysM domains of bacterial origin bind peptidoglycan fragments and chitin polymers with similar affinity, although plant LysM demonstrate a greater affinity for chitin [21]. Similarly, a protein consisting of the three LysM domains from the *Lactococcus lactis* autolysin AcaA binds both bacterial-derived peptidoglycan sacculus as well as chitin and the cell wall of the fungus *Psilocybe cubensis* [22]. In vivo, the LysM domain-containing *A*. *thaliana* kinase CERK1 responds to both peptidoglycan and chitin [23] and the rice LysM receptor-like kinase CERK1 [24] and LysM-domain containing proteins LYP4 and LYP6 [25] respond to chitin as well as to peptidoglycan.

**Table 1:** Chitin/PG biding of LysM-domain containing proteins. When the specific comparative specificity has been determined, it is noted; otherwise ligands with approximately comparable affinities observed are listed.

Organism	LysM-domain proteins	Specificity
*E*. *faecalis*	AtlA	Chitin>PG [6]
*L*. *lactis*	AcaA	Chitin, PG [22]
*M*. *smegmatis*	MSL	Chito-oligosaccharide, Chitin, PG [39].
*B*. *subtilis*	NlpC/P60	Chitin, PG [21]
*T*. *atroviride*	TAL6	Chitin, PG [40]
*A*. *thaliana*	CERK1	PG [23], chitin [23]
*O*. *sativa (rice)*	OsCERK1, OsLYP4, OsLYP6	Chitin/PG [24], [25]
*M*. *japonicus (shrimp)*	MjLPBP	Chitin, PG [41]

**Abbreviations:** PG, peptidoglycan.

So how is specificity in signaling maintained? One possibility is some kind of post-translational modification to the LysM domain (e.g., *N*-glycosylation [26]) that differentially affects binding of different GlcNAc-containing molecules. Alternatively, modifications could be targeted to the ligands. Peptidoglycan is often modified, and these modifications can affect its interaction with the innate immune system [27]. For example, wall teichoic acids of *Staphylococcal aureus* affect binding to an important peptidoglycan recognition protein in *Drosophila melanogaster* [28], although whether these or other modifications affect LysM binding is not known. Structural studies of chitin binding to a protein containing multiple LysM domains suggest that cooperative binding to the GlcNAc strand could be important [21, 29]. And in the case of peptidoglycan, multiple LysM domains increase the affinity, at least as compared to chito-oligosaccharides [6]. However, while these and other studies implicate the length of the saccharide polymer as an important factor in specificity, the size of the chitin or peptidoglycan fragments that are physiologically active is not known [30, 31]. While very small peptidoglycan fragments derived from the gut flora can exogenously induce the genesis of lymphoid follicles in mice [32], similar molecules do not stimulate immunity-associated defenses in *Arabidopsis* [23]. Thus, polymer chain length is likely a critical but not well-understood parameter for specificity of activation.

### Promiscuity of carbohydrate ligands in pathologies

While the mechanisms underlying specificity discussed above may be sufficient for normal physiological function, the presence of aberrantly higher levels of one class of carbohydrate could interfere with these mechanisms. That is, during a bacterial infection or environmental exposure to fungal-derived [33] or insect-derived chitin [2], the relative concentrations of ligands may undergo significant changes with potential pathological consequences. Chitin is thought to be an important trigger leading to the development of asthma. Chitin administered to murine airways induces infiltration of eosinophils and basophils and drives stimulation of both resident and recruited macrophages [34]. In addition, mammalian lung epithelial cells secrete a chitinase, and mice lacking this enzyme exhibit premature morbidity and mortality, concomitant with significant airway accumulation of environmentally derived chitin polymers [35]. One possible explanation for this pathology is that increased chitin levels result in aberrant activation of the peptidoglycan receptors in the lung, thereby disturbing the normal balance of ligand concentration required to achieve proper specificity of recognition. Although the specific peptidoglycan receptors in the lung are not known, LysM domain-containing proteins of unknown function are found in mammals [10]. Promiscuous activation could also be relevant to the intriguing but not well-understood role of the microbiota in the pathogenesis of asthma [36]. Recent observations that transient early life microbial dysbiosis is an important factor influencing asthma development [37] suggest that bacterial products may have a role in mediating these effects. Specifically, peptidoglycan is released by growing bacterial cells, and the substantial levels of peptidoglycan fragments generated by the microbiota have important systemic immunological effects mediated by key innate immune proteins like Nod1 [30]. Thus, perhaps the levels of these molecules relative to their receptors is affected by the presence of chitin, which changes the normal ligand:receptor stoichiometry. This would be analogous to the situation in plants in which exogenous Nod factors can act as a competitive agonist and suppress the normal plant innate immune reaction to chitin [14]. Finally, inflammatory bowel disease is another example in which interactions between chitin- and peptidoglycan-signaling systems could be relevant. Recent work has demonstrated that chitin microparticles administered to colitis models greatly affected the bacterial community in the colon, both in overall number as well as overall species composition [38].
